# New acute respiratory management in tracheal rupture caused by chest trauma

**DOI:** 10.1186/s13019-020-01298-6

**Published:** 2020-09-12

**Authors:** Cheng Shen, Lin Ma

**Affiliations:** Department of Thoracic Surgery, West-China Hospital, Sichuan University, Chengdu, 610041 China

**Keywords:** Chest trauma, Tracheal rupture, Mechanical ventilation, Emergency surgery

## Abstract

We report a case who is a 33-year-old man admitted to our Emergency room for chest trauma caused by the factory’s mechanical arm. Despite the endotracheal tube, the patient’s respiratory state was poor and the oxygen saturation did not improve and the subcutaneous emphysema progressed. To improve distressed breathing, we first proposed the concept “mechanical ventilation with dual ventilator” to maintain oxygen saturation of the patient. This is, to our knowledge, the first report of using a special mechanical ventilation method in emergency surgery.

A 33-year-old man was admitted to our Emergency Room for chest trauma caused by the factory’s mechanical arm. He was a non-smoker. Physical examination revealed noticeable twist feeling under the skin of his neck, chest and abdomen. Plain chest computed tomography (CT) revealed suspected damage to the right tracheal membrane of patient (Fig. 1a and b, red arrow) and multiple fractures of the bilateral ribs, including the anterior branch of the left first and second ribs, and the posterior branch of the ribs in the right side (3th, 5th, 6th and 7th). It also showed soft tissue swelling and subcutaneous emphysema in neck, anterior chest wall, back and abdomen. Mediastinum emphysema and bilateral lung contusion were existing in chest CT obviously. On both sides of the chest, pneumothorax with pleural effusion and bilateral drainage tube shadows were seen in chest images. His oxygen saturation level decreased to 80%. However, due to the unstable condition of the patient and short time (less than 45 min) for organizational reason in Emergency room, especially the mediastinum and subcutaneous emphysema of the whole body were aggravated in short time, there was no more time to arrange bronchoscopy and gastroscopy.

**Fig. 1:**
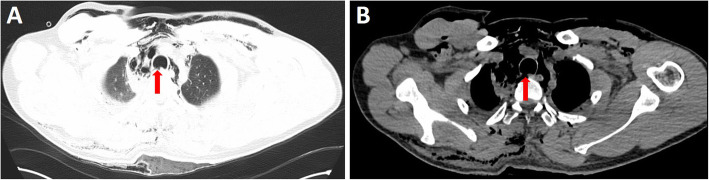
Plain chest CT revealed suspected damage to the right tracheal membrane of patient (red arrow)

We approached the right chest via the thoracotomy with general anesthesia under double-lumen tube mechanical ventilation immediately. The patient’s respiratory state was poor, and gas analysis of the arterial blood revealed a pH of 7.29, pO_2_ of 50 mmHg, pCO_2_ of 51 mmHg, and 65% O_2_ saturation during the operation. Despite the endotracheal tube, the oxygen saturation did not improve and the subcutaneous emphysema progressed. Then we check the contralateral chest cavity through the anterior mediastinum to exclude the left pneumothorax for the suspicion on the chest tube occlusion. The main reason for poor oxygen saturation is severe left lung contusion. During intraoperative exploration and auxiliary observation by bronchoscope, we found that the tearing of tracheal membrane from the top of the right thorax down to the carina near the right main bronchus, and a 0.5*0.5 cm cavity was seen in the carina membrane. A diagnosis of tracheal rupture was made during the surgery. At the same time, as the patient was in the left lateral position during the operation, extracorporeal membrane oxygenator (ECMO) could not be placed. To improve distressed breathing, we proposed the concept “mechanical ventilation with dual ventilator” to maintain oxygen saturation of the patient. Specifically, a 0.5 cm incision was made at the right intermediate bronchus, and a single lumen tube (5#) was inserted to maintain ventilation of the right middle and lower lobes (Fig. 2). The PEEP, FiO2, tidal volumes and respiratory pressure of left lung were 6 cmH2O, 90%, 162 ml (6 ml/kg*60 kg*45%) and 20 cmH2O respectively. The PEEP, FiO2, tidal volumes and respiratory pressure of right lung were 5 cmH2O, 90%, 108 ml (6 ml/kg*60 kg*30%) and 20 cmH2O respectively. The oxygen saturation of patient was significantly improved, and maintained around 98%. During the operation, free myocutaneous flap of the right posterior chest wall pleura was formed to cover the defect of the carina (At the level T4 of the anastomosis, insert a pedicled pleural flap with the proximal trachea side not to be broken, approximately 4 × 8 cm in size, and wrap the flap around the carina from back to front), and the remaining tracheal membrane was sutured with 3–0 sutures to repair the fracture. The patient was discharged 7 days after the operation with no complication.

**Fig. 2:**
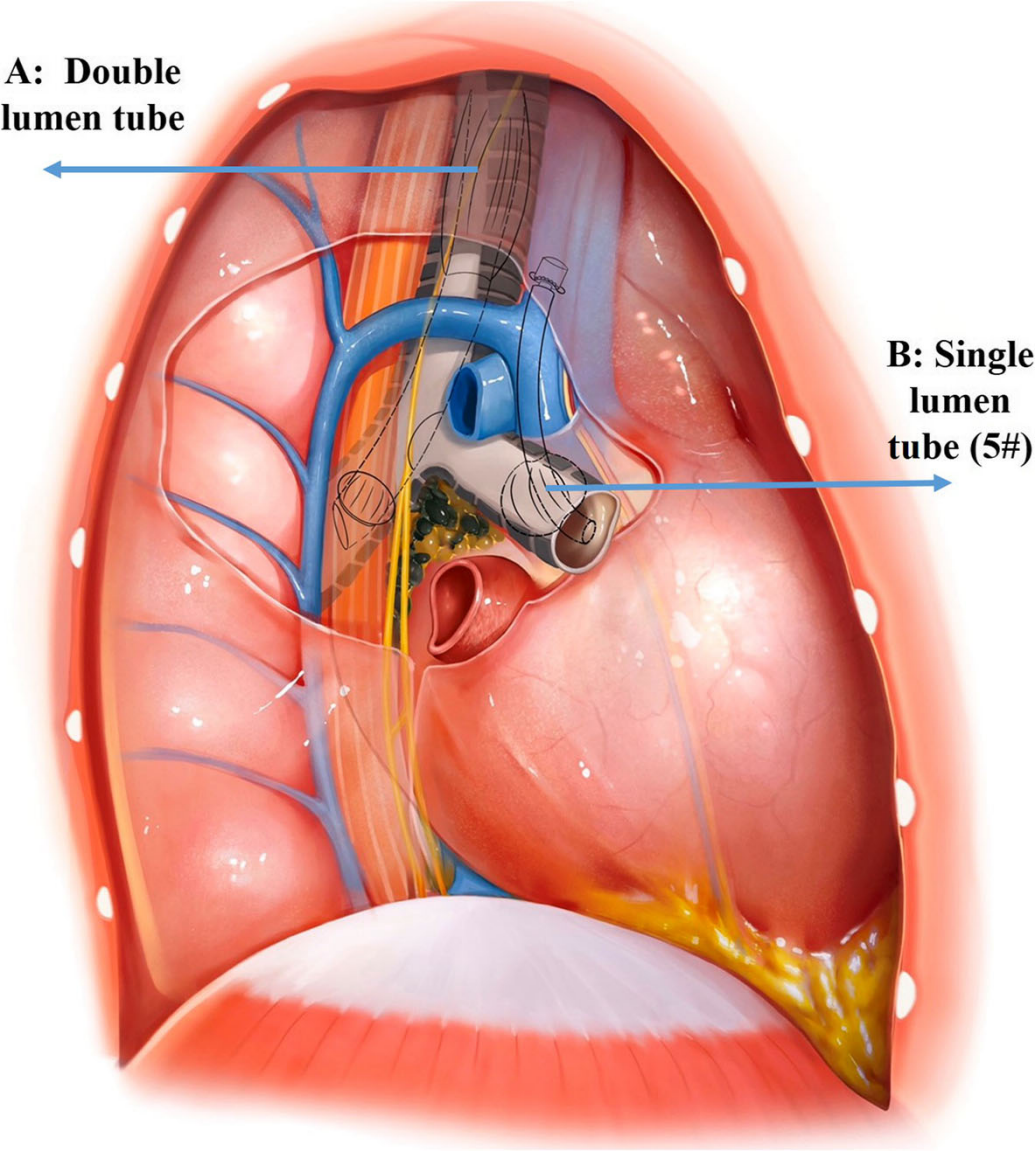
The concept “mechanical ventilation with dual ventilator”. **A** is the double-lumen tube which maintains oxygen saturation of the patient. A 0.5 cm incision was made at the right intermediate bronchus, and a single lumen tube (5#) (**B**) was inserted to maintain ventilation of the right middle and lower lobes

This is, to our knowledge, the first report of using a special mechanical ventilation method in emergency thoracic surgery. Tracheobronchial rupture from chest trauma is a potentially lethal injury [1]. In some cases, patients are not in a condition to safely perform bronchoscopy in emergency rooms [2, 3]. “Independent lung ventilation” has been used for some time in the ICU or operation room for regular surgery or ARDS [4–6].

In patients with mechanical ventilation, the cuff of the single lumen tube was placed distal of the laceration with sufficient reserve to the carina. If the laceration affected the tracheal bifurcation or the right or left main stem bronchus, the opposite bronchus was intubated with a corresponding double-lumen tube [7, 8]. However, in our case, the patients was intubated with double-lumen tube, but the left single lung ventilation cannot be maintained in surgery. So we proposed innovative ventilation method called “mechanical ventilation with dual ventilator”. The reasons for the application include the critical condition of the patient without the result of bronchoscopy and gastroscopy before the operation. Secondly, the poor single lung ventilation cannot be maintained in surgery and pneumothorax should be excluded; the application of ECMO in the lateral position during thoracic surgery also faces difficulties. The application of the “mechanical ventilation with dual ventilator” is simple, practical and efficient method, which effectively solves the state of hypoxic saturation of patients, and it can be applied in a timely manner in future emergency thoracic surgery.

## Data Availability

All data for this study are publicly available and are ready for the public from database of hospital.
